# Establishment and characterization of a novel *MYC/BCL2* “double-hit” diffuse large B cell lymphoma cell line, RC

**DOI:** 10.1186/s13045-015-0218-1

**Published:** 2015-10-29

**Authors:** Lan V. Pham, Gary Lu, Archito T. Tamayo, Juan Chen, Pramoda Challagundla, Jeffrey L. Jorgensen, L. Jeffrey Medeiros, Richard J. Ford

**Affiliations:** Department of Hematopathology, The University of Texas MD Anderson Cancer Center, 1515 Holcombe Blvd. Unit 54, Houston, TX 77030 USA

**Keywords:** Double-hit lymphoma (DHL), DLBCL, RC cell line, *MYC*, *BCL2*

## Abstract

**Background:**

Diffuse large B cell lymphoma (DLBCL) is the most common type of lymphoid malignancy worldwide. Approximately 5 % of cases of DLBCL are so-called double-hit lymphomas (DHL), defined by a chromosomal translocation or rearrangement involving *MYC*/8q24.2 in combination with another recurrent breakpoint, usually *BCL2*/18q21.3. Patients with *MYC/BCL2* DHL are resistant to standard front-line therapy, and currently, there is no consensus for a therapeutic strategy to treat these patients. Lack of clinically relevant or validated human experimental DHL models of any type that would improve our understanding of the biologic basis of *MYC/BCL2* DHL pathophysiology continues to hamper identification of valid therapeutic targets. We describe a unique *MYC/BCL2* DHL cell line with morphologic features of DLBCL that we have established, designated as RC.

**Methods:**

We used tissue culture techniques to establish the RC cell line from primary DLBCL cells. We also utilized molecular and cellular biological techniques including flow cytometry, polymerase chain reaction (PCR), DNA fingerprinting, reverse-phase protein array, conventional cytogenetics, and fluorescence in situ hybridization (FISH) analysis to characterize the RC cell line. NSG-severe combined immunodeficiency (SCID) mice were utilized as a model for xeno-transplantation of RC cells.

**Results:**

RC cells had the following immunophenotype: positive for CD10, CD19, CD20, CD22, CD38, CD43, CD44, and CD79b and negative for CD3, CD4, CD5, CD8, CD11c, CD14, CD30, CD56, and CD200, which was identical to the primary tumor cells. Conventional cytogenetic analysis showed a t(2;8)(p12;q24.2) and t(14;18)(q32;q21.3), corresponding to *MYC* and *BCL2* gene rearrangements, respectively. DNA fingerprinting authenticated the RC cell line to be of the same clone as the primary tumor cells. In addition, RC cells were established in SCID mice as an in vivo model for translational therapeutics studies. Proteomic analysis showed activation of the mTOR signaling pathway in RC cells that can be targeted with an mTOR inhibitor.

**Conclusion:**

The data presented confirm the validity of the RC cell line as a representative model of *MYC/BCL2* DHL that will be useful for both in vitro and in vivo studies of DHL pathogenesis and therapeutics.

## Background

Diffuse large B cell lymphoma (DLBCL) is the most common type of non-Hodgkin lymphoma worldwide. In the USA, DLBCL represents approximately 30 % of all new lymphoma cases per year and is the fifth most common cancer [1]. Current standard front-line therapy for DLBCL patients involves rituximab immunotherapy and cyclophosphamide, doxorubicin, vincristine, and prednisone (R-CHOP). Approximately 70–80 % of patients experience some form of remission, but relapsed/refractory DLBCL occurs in 30–40 % of patients within 2–3 years, and this patient subset has poor salvage therapy options [1–4].

DLBCL is a molecularly heterogeneous disease [5, 6]. Approximately 30–40 % of cases of DLBCL are characterized by recurrent chromosomal translocations involving *BCL6*/3q27, *BCL2*/18q21.3, and *MYC*/8q24.4 in about 30, 20, and 10 % of DLBCL cases, respectively. In recent years, the concept of double-hit lymphoma (DHL) has received much attention in the literature. DHL is defined by a chromosomal breakpoint affecting the *MYC*/8q24.2 locus in combination with another recurrent oncogene breakpoint, usually *BCL2* and less often *BCL6* or rarely other genes. *MYC/BCL2* DHL represents approximately 70 % of all cases of DHL. Double-hit lymphoma (all types) represents about 5 % of all cases of DLBCL and affected patients generally have an aggressive clinical course with poor prognosis, despite combination chemotherapy, with a median overall survival less than 1–2 years [7].

To date, exploratory studies to determine the pathogenesis of DHL have been limited, in part due to the lack of a validated lymphoma cell model that is both immunophenotypically and genetically consistent with the original primary DHL tumor. To our knowledge, there have been only a small number of published manuscripts demonstrating the establishment and characterization of defined DHL cell lines. The CJ cell line that we established in 1990 before recognition of the clinical importance of DHL is believed to be the first DHL cell line showing both *MYC* and *BCL2* gene rearrangements [8]. In 2003, we established another *MYC/BCL2* DHL cell line, designated EJ-1, that morphologically resembled DLBCL [9], and recently, Hooper et al. [10] described the establishment of a novel *MYC/BCL2* DHL cell line, U-2973. Several recent studies indicate that the OCI-LY18, Sc-1, and CARNAVAL DLBCL cell lines also appear to demonstrate *MYC/BCL2* double-hit characteristics [11, 12], but a comprehensive genetic analysis of these cell lines has not been published. Collectively, these cell lines should provide excellent models to study the pathophysiology and translational biology of *MYC/BCL2* DHL. However, because these cell lines were never genetically authenticated against the primary tumor, the exact origin of these cells remains unclear. Thus, additional, validated DHL cell lines are a prerequisite for increasing our understanding and therapeutic potential of DHL.

Herein, we described the establishment and characterization of a novel *MYC/BCL2* DHL cell line with morphologic features of DLBCL, designated RC, that closely shares an immunophenotype and cytogenetic features of the primary B cell tumor at diagnosis.

## Results

### Establishment of the RC cell line

**Fig. 1:**
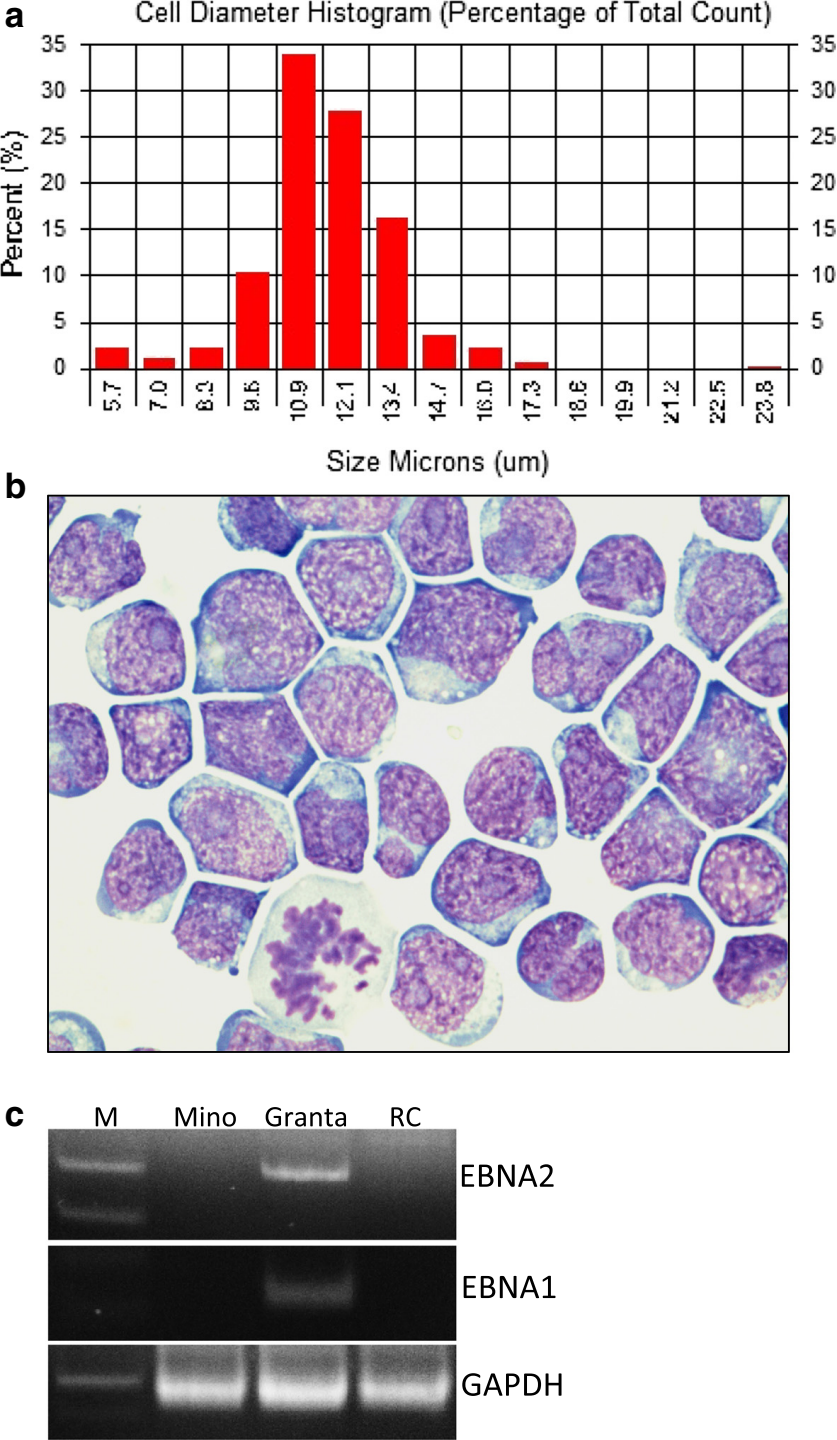
Morphologic and phenotypic features of RC cells. **a** Distribution of the size (longest diameter) of RC cells after 16 months of cell culturing. **b** Representative image of H&E-stained RC cells after 16 months in cell culture. **c** PCR analysis for EBV type 1 (EBNA1) and type 2 (EBNA2) gene in Mino (negative control), Granta (positive control), and RC cell lines. GAPDH serves as a loading control

Primary cells were obtained from a pleural effusion of a patient diagnosed with diffuse large B cell lymphoma with high-grade features (high mitotic activity and proliferation rate). The primary cells were washed, explanted, and cultured at approximately 5 × 10^6^ cells/mL in RPMI-1640 media, supplemented with 15 % fetal bovine serum (FBS) without any external stimulation. The primary cells remained viable (~90–95 %) even after 4 weeks in cell culture; however, the number of cells remained constant. During the fifth week in culture, cell number began to increase and identifiable mitotic figures began to appear. From this timepoint, the cells doubled in number every 4–5 days. This established lymphoma cell line successfully continued cell proliferation in a single-cell suspension without cellular clump formation, growing in continuous culture for more than 16 months, and aliquot samples could be frozen in medium composed of 90 % FBS and 10 % DMSO. The cell line was designated as the “RC” cell line, optimally maintained at a density between 1 and 2 × 10^6^ cells/mL and could be split 1:2 every 3–4 days. RC cells are medium-to-large, blast-like lymphoid cells, approximately 9–14 μm in largest diameter (Fig. 1a) with moderately abundant strongly basophilic cytoplasm. The nuclei were round to ovoid with coarse chromatin and occasional irregular nuclear contours. The morphologic features of RC cells were stable and did not change during 16 months in culture (Fig. 1b).

### EBV status in RC cells

The RC cells were shown to be negative for Epstein-Barr virus (EBV) by polymerase chain reaction (PCR) analysis. Epstein-Barr viral genomes (types 1 and 2) were detected in the immortalized Granta MCL cell line (positive control) but not in the MCL Mino cell lines (negative control) as expected (Fig. 1c).

### Immunophenotypic characterization of RC cells by flow cytometry

**Fig. 2:**
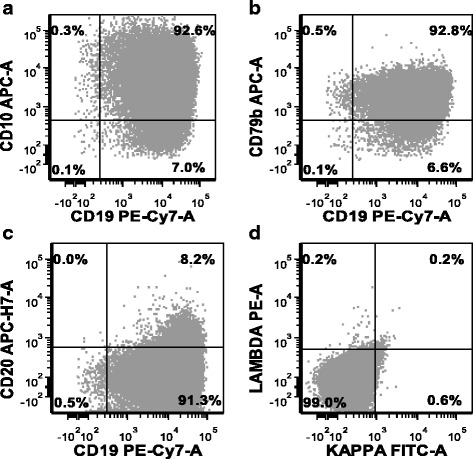
Immunophenotype of RC cells. Representative flow cytometric histograms of total RC cells. **a** RC cells are positive for CD19 and CD10; **b** they are positive for CD79b, implying surface immunoglobulin expression; **c** they are positive for CD20 in only a small subset; **d** they stained negative for both surface kappa and lambda Ig light chains

We performed flow cytometry immunophenotypic analysis on the original pleural effusion sample and later on the established RC cell line after 12 months of continuous culture. The immunophenotypes of the primary tumor cells and RC cells were virtually identical (Table 1). RC cells are positive for CD10, CD19, CD20 (small subset), CD22, CD23, CD38, CD43, CD44 (partial), CD45, and CD79b and are negative for CD3, CD4, CD5, CD8, CD11c, CD14, CD30, CD200, or surface kappa and lambda light chains. Representative flow cytometry histograms showing that 99 % of RC cells are positive for CD10, CD19, and CD79b, with no staining for surface kappa and lambda light chains are shown (Fig. 2). However, the presence of surface CD79b implies surface immunoglobulin expression and therefore suggests that RC cells express an Ig light chain that was not detectable by our standard anti-kappa or anti-lambda monoclonal reagents. The original effusion cells showed no staining for CD20, possibly due to blockade by recently administered rituximab (anti-CD20) therapy. The RC cell line showed very dim expression of CD20 (compared with normal mature B cells), possibly due to selection of primary lymphoma cells with low expression. Alternatively, dim or absent CD20 expression has been reported in cases of DHL by others [13]. RC cells also express a high level of CD38, an indicator of MYC rearrangement [14].

**Table 1:** Immunophenotype profile of primary DH-DLBCL cells and RC cells as determined by flow cytometry

Phenotype	Primary cells	RC cells
CD5	−	−
CD10	+	+
CD19	+	+
CD20	−	+/−
Kappa light chain	−	−
Lambda light chain	−	−
CD79b	+	+
CD23	+	+
CD43	+	+
CD44	+/−	+/−
CD45	+	+
CD11c	−	−
CD200	−	−
CD22	+	+
CD3	−	−
CD4	−	−
CD8	−	−
CD14	−	−
CD30	−	−

− negative staining, + positive staining, +/− dim/partial staining

### Conventional cytogenetics and FISH analysis

**Fig. 3:**
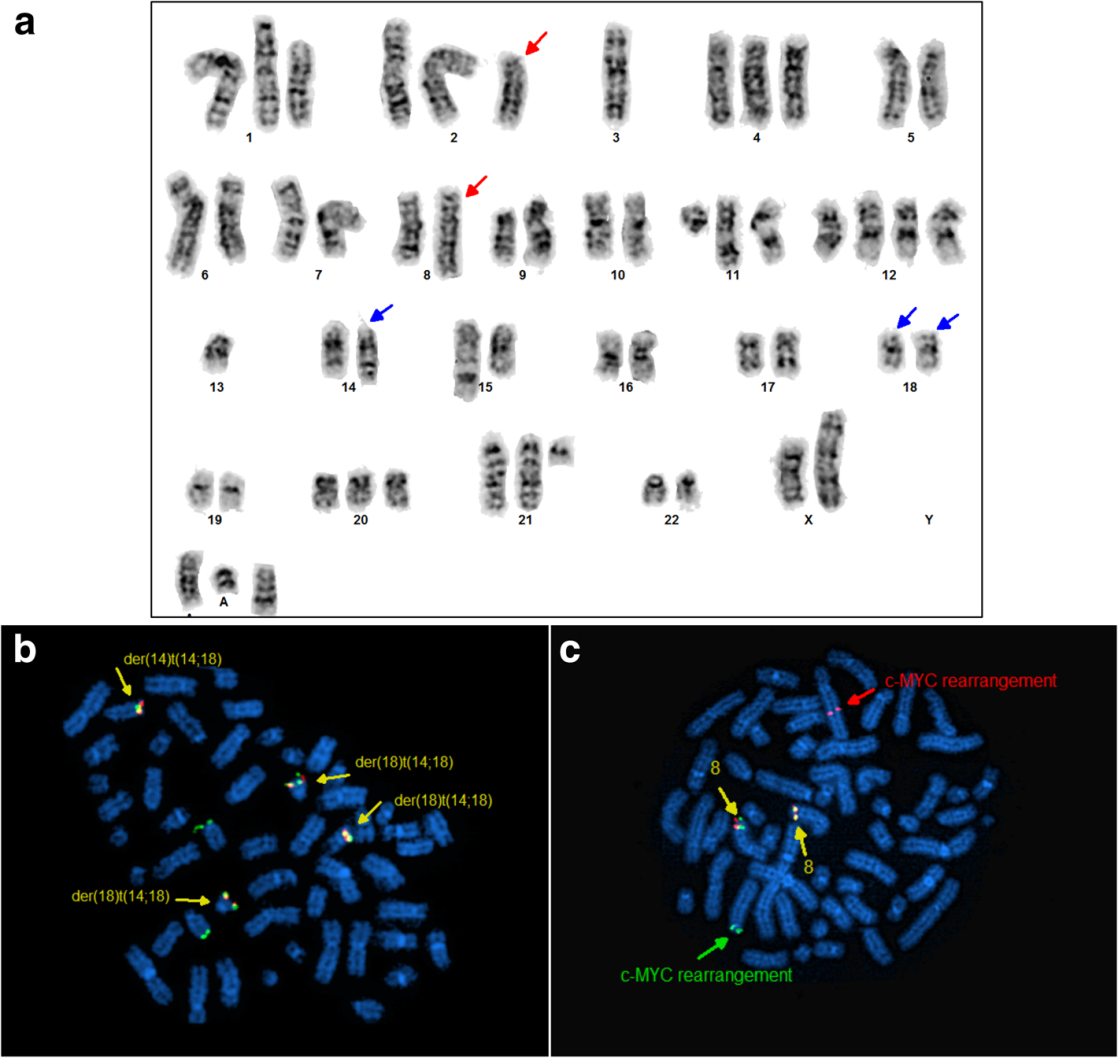
Conventional cytogenetics and FISH analysis of RC cells. **a** Representative karyotype of RC cells. *Red arrows* point to t(2;8)(p12;q24.2) translocation, and *blue arrows* point to t(14;18)(q32;q21.3) translocation also showing an extra copy of der(18)t(14;18) in the karyotype. **b** FISH analysis confirmed t(14;18) in RC cells. *IGH/BCL2* dual-color, dual-fusion translocation probes were used. *Green*: probe for the immunoglobulin heavy chain gene; *red*: probe for the BCL2 gene; *yellow*: *IGH-BCL2* fusion gene signal. **c** FISH analysis of *MYC* gene rearrangement, showing one *red* and one *green* on the der(8)t(2;8) and der(2)t(2;8), respectively

Conventional cytogenetic analysis of RC cells revealed a complex karyotype, including a t(2;8)(p12;q24.2) and a t(14;18)(q32;q21.3) (Fig. 3a). The karyotype of RC cells is similar to, but more complex than, the karyotype of the primary lymphoma cells (Table 2). However, only 15 metaphases were available for analysis of the primary tumor cells. Interphase fluorescence in situ hybridization (FISH) analysis with FISH mapback performed on the RC cells showed multiple copies of *IGH/BCL2* fusion gene (Fig. 3b) and an allelic *MYC* rearrangement most likely involving the *IGK* gene mapped to 2p12 region (Fig. 3c) and corroborating the t(2;8)(p12;q24.2) identified by conventional cytogenetic analysis (Fig. 3a). RC cells were negative for *IGH/MYC* and *IGH/CCND1* fusions as assessed by FISH (data not shown).

**Table 2:** Clonal cytogenetic abnormalities in lymphoma cells from primary bone marrow sample and from RC cell line

Karyotype	
Primary cells	RC cell line
45~56,X,der(X)t(X;3)(p22.1;p13),+1,add(1)(p32), i(1)(q10),-3,del(3)(p13p25),add(6)(q23),+7,der(8) t(2;8)(p12;q24.2),t(11;15)(q13;q26),+12,t(14;18) (q32;q21.3),+21,+2~6mar[cp9]/46,XX[6]	45~56,X,der(X)t(X;3)(p22.1;p13),+1,add(1)(p32),del(1)(p34.3p36.1),t(2;8) (p12;q24.2), −3,+4, add(6)(q23), +8,+11,t(11;15)(q13;q26), +12,+12,−13, add(13)(q34), t(14;18)(q32;q21.3), +der(18)t(14;18)x1~2, +20,+21, psu dic(21;1)(q22;p13)x2, +2~4mar[cp20]

### STR DNA fingerprinting analysis of primary cells and RC cells

Using a multiplex short tandem repeat (STR) DNA fingerprinting system, which allows for the detection of unique DNA fingerprints through the genotyping of 16 STR loci, primary lymphoma cells and the RC cells shared 100 % identity. These results confirm that the genetic parentage of RC cells is, in fact, the patient’s lymphoma cells (Table 3). The RC cell line profile did not match any other cell line profile in the current database at MD Anderson Cancer Center or elsewhere.

**Table 3:** STR DNA fingerprinting of primary cells and RC cells

Sample	STR Loci							
	AMEL	CSF1PO	D13S317	D16S539	D21S11	FGA	THO1	TPOX
Primary cells	X,Y	10,12	12	11	28,32.2	21,24	9,9.3	8,11
RC cells	X,Y	10,12	12	11	28,32.2	21,24	9,9.3	8,11

### Xeno-transplantation of RC cells into SCID mice

**Fig. 4:**
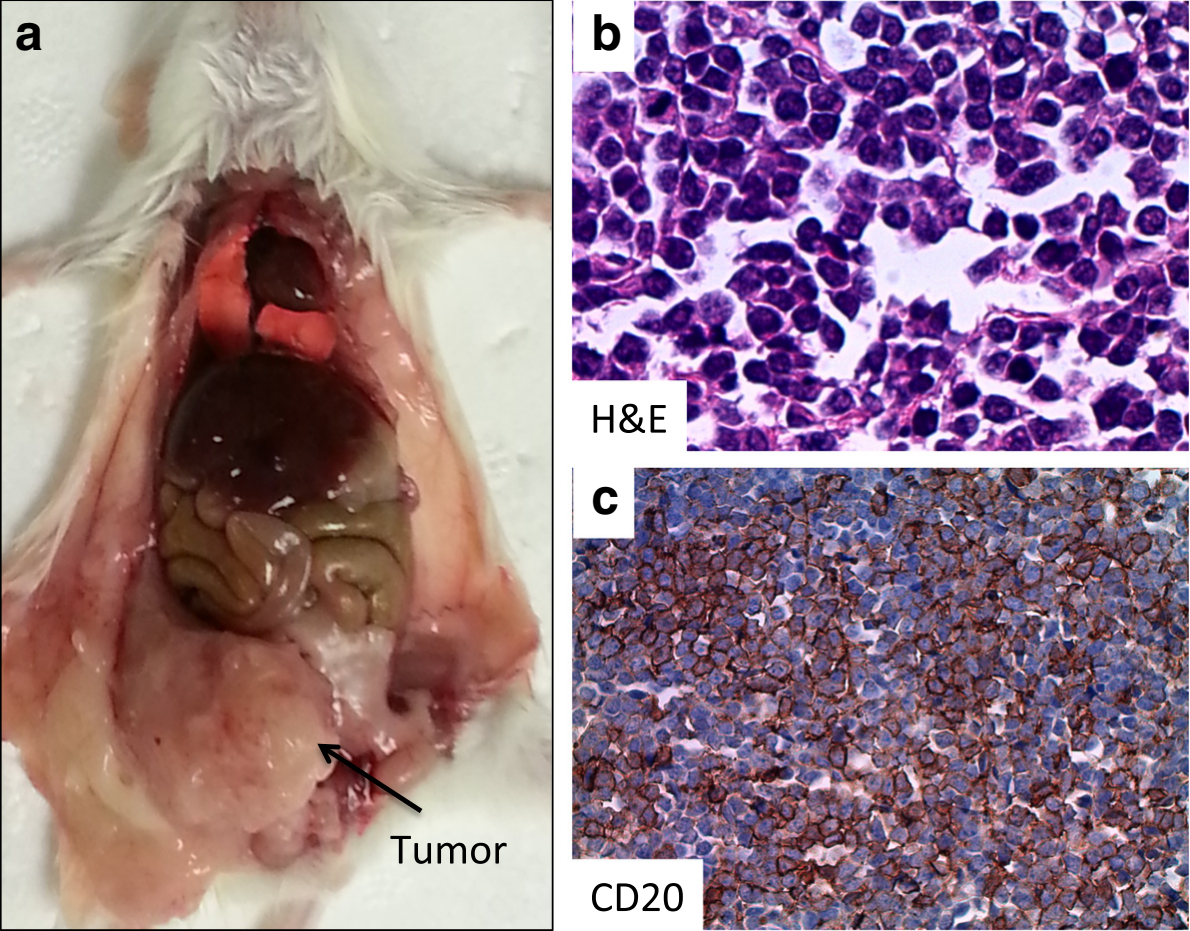
Xeno-transplantation of RC cells into SCID mice. **a** Necropsy of a SCID mouse that received a transplant of RC cells. *Arrow* pointing to the tumor mass. **b** H&E paraffin sections of the SCID peritoneal tumor bearing the RC tumor cells. **c** Immunohistochemical analysis of the transplanted lymphoma from a SCID mouse showing the expression of CD20

We inoculated RC cells by intraperitoneal (IP) injection into the lower abdomen of five severe combined immunodeficiency (SCID) mice. Within 5 weeks, all five (100 %) mice developed a solid tumor mass in the lower abdomen (Fig. 4a). Further analysis showed that the excised tumor mass was composed of lymphoid cells (Fig. 4b) of human B cell origin, CD20 positive with characteristic CD20 cell membranous expression (Fig. 4c), morphologically consistent with RC cells.

### Reverse-phase protein array (RPPA) analysis and response to an mTOR inhibitor

**Fig. 5:**
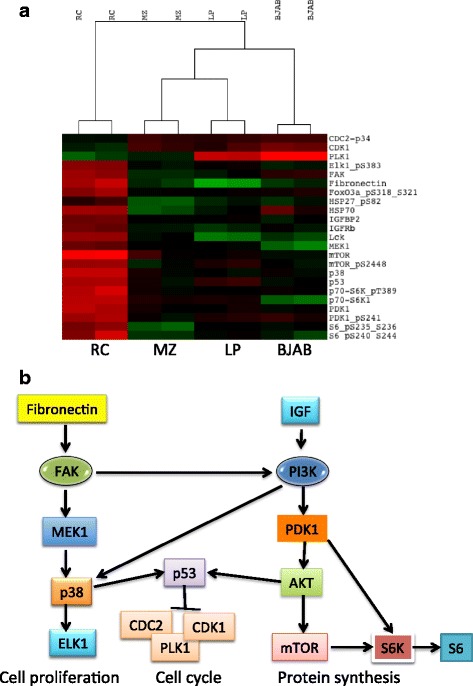
Reverse-phase protein array analysis of RC-DHL cell line. **a** Supervised hierarchical clustering heatmap of 285 proteins demonstrating the differential protein expression profile of RC cells in comparison to three representative non-DHL-DLBCL cell lines, MZ, LP, and BJAB. **b** Schematic diagram showing the potential pathways activated in RC cells based on the RPPA analysis

We performed RPPA analysis on the RC cell line and three additional DLBCL cell lines to determine the protein signaling profile of RC cell line. After quality control analysis, expression data for 285 proteins were available for further analysis. Supervised hierarchical clustering of the data for all proteins showed a set of up-regulated and down-regulated proteins in the RC cell line that differed from the other three DLBCL cell lines (Fig. 5a). Based on the RPPA data, a diagram model was constructed showing activation of the integrin-MEK-ELK1 and the insulin-AKT-mTOR signaling pathways as being used prominently in RC cells (Fig. 5b).

**Fig. 6:**
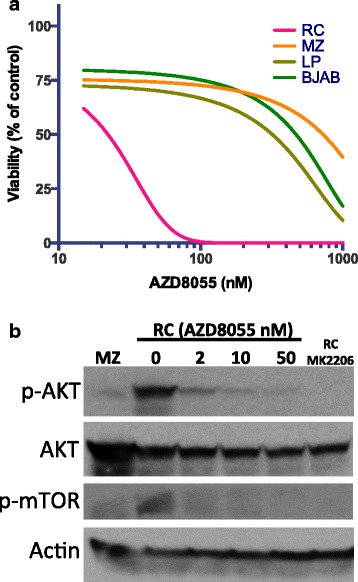
Effects of an ATP competitive mTOR inhibitor AZD8055 in RC cells. **a** Cells from RC and three additional representative DLBCL cell lines (MZ, LP, and BJAB) were treated with increasing concentrations of AZD8055. Cell viability was assessed by the CellTiter-Glo Luminescent Assay after 72 h of treatment. Graph shows data that is representative of two independent experiments with triplicate wells for each drug concentration. **b** RC cells were treated with the indicated AZD9055 concentrations for 24 h. MZ cells were used as a negative control. RC cells treated with the AKT inhibitor MK-2206 were also used as a control. Cell extracts were purified and immunoblot for p-AKT, AKT, and p-mTOR. Actin was used a loading control

We also used a small molecule inhibitor of mTOR (AZD8055) to determine whether RC cells can respond to this agent. As demonstrated in Fig. 6a, RC cells are highly sensitive to AZD8055 in comparison to the other three DLBCL cell lines. In addition, RC cells treated with AZD8055 down-regulate protein expression of phosphor-AKT and phosphor-mTOR that are low or absent in AZD8055-resistant MZ cells (Fig. 6b). These findings indicate that the RC cell line is an excellent model for identifying potential therapeutic agents, targeting pathways like the PI3K-ATK-mTOR survival pathway.

## Discussion

Although others had reported cases of DLBCL with *MYC* and *BCL2* rearrangements previously [17–20], Aukema and colleagues [16] in 2011 published an important review article that introduced the concept of DHL. Aukema and colleagues defined DHL as a neoplasm characterized by a *MYC* rearrangement combined with another genetic abnormality, such as *BCL2*, *BCL3*, *BCL6*, or other genes. Currently, over 400 cases of DHL have been reported in the literature, with the combination of *MYC* and *BCL2* being, by far, the most common. These studies have shown that patients with double-hit lymphoma associated with *MYC*/8q24.2 and *IGH-BCL2*/t(14;18) have an aggressive disease, clinically characterized by B type symptoms, advanced clinical stage, a high International prognostic index (IPI), poor response to standard front-line R-CHOP or more aggressive therapies, and a very poor prognosis with a median survival of 1–2 years [7, 21]. As a result of the poor prognosis, DHL is currently a subject of intense clinical and research interest because there is no consensus therapeutic approach for these patients and the conceptual/mechanistic basis underlying the DHL remains unclear [15].

A major limitation to the successful treatment of patients with *MYC/BCL2* DHL is an improved understanding of disease pathogenesis, mechanisms of chemotherapeutic resistance, and knowledge of potential therapeutic targets for which new therapies can be rationally designed. We suggest that the RC cell line reported here is of interest and will be a useful tool that will be helpful in contributing to an improved understanding of *MYC/BCL2* DHL. The RC cell line has the advantage of having been well studied initially, with further relevant follow-up studies. Its derivation from a patient with a DHL is clearly identified by STR analysis. The RC cells have usual morphologic features of DLBCL and the *MYC* and *BCL2* abnormalities are well documented by conventional cytogenetic analysis and fluorescence in situ hybridization (FISH). The *MYC* rearrangement involves *IGK* gene mapped at 2p12 region corroborating the t(2;8)(p12;q24.2) identified by constitutional cytogenetic analysis. RC cells have a germinal center B cell immunophenotype, as is the case for almost all published cases of *MYC/BCL2* DHL, and a complete immunophenotype is shown by flow cytometry immunophenotypic analysis. Similar to the original lymphoma cells, RC cells showed dim/low CD20 expression. The molecular mechanism(s) resulting in decreased expression of CD20 in RC cells and in DHL are unclear and have not been explored [13]. The decreased expression of CD20 in DHL suggests that the use of second-generation monoclonal antibodies targeting CD20 may be fruitful because these engineered antibodies are reportedly more effective than rituximab in inducing complement-dependent cytotoxicity, particularly in tumors with decreased CD20 antigen density [22, 23].

Several general findings have emerged from recently published DHL retrospective series [7]. These studies show that patients with DHL often present with extranodal disease, central nervous system (CNS) involvement is more common, and higher international prognostic index (IPI) scores. However, retrospective studies have not been able to contribute to a deeper understanding of DHL or provide clues to potential therapeutic targets that would enable substantial progress in therapy. Using RPPA analysis, we have identified at least two important growth/survival pathways (integrin-MEK-ELK1 and the insulin-AKT-mTOR) that are highly activated in RC cells. RC cells are highly sensitive to small molecule inhibitors of the AKT-mTOR pathway. Although it has already been shown that the PI3K/Akt/mTOR pathway is highly active in many B cell malignancies, including DLBCL [24], our study is the first to demonstrate the activation of these growth/survival pathways in a representative DHL-DLBCL cell line. However, a recent study showed that the PI3K/mTOR inhibitor BEZ235 can potentiate the activity of the HDAC inhibitor panobinostat in pre-clinical models of DLBCL, including DHL cell lines with overexpression of bcl-2 and MYC [12], further suggesting activation of Akt/mTOR activation in DHL. Further studies in more DHL cell lines as well as primary cells are required to validate whether the integrin-MEK-ELK1 and the insulin-AKT-mTOR pathways are commonly activated and can be targeted in DHL. Targeting the PI3K/mTOR pathway as was shown in this study is just one example of the utility of the RC cell line in biomarker research and drug development. New drugs, particularly targeted therapeutic agents [25–27], are increasingly being developed and entered the clinic in recent years. Therefore, a fully characterized *MYC/BCL2* DHL cell line with morphologic features of DLBCL, like RC, will be valuable for researchers in identifying novel targets and pre-clinical screening studies of novel therapies that potentially can benefit patients [28].

Although not a specific focus of this study, it seems that the concept of DHL has some limitations. It appears likely that disease and resistance mechanisms in *MYC/BCL2* DHL are likely to differ from *MYC/BCL6* DHL and therefore the designation of DHL is descriptive but not sufficiently specific. Even with the most common *MYC/BCL2* DHL, one of our early cell lines, CJ, was derived from an elderly woman with typical low-grade follicular lymphoma, with the usual t(14:18)(q32;q21.3) who was initially successfully treated with conventional CHOP chemotherapy, achieving a remission lasting several years. This patient subsequently relapsed with aggressive *MYC/BCL2* DHL with a complex karyotype and multiple other uncharacterized cytogenetic abnormalities. Interestingly, this DHL did not show the expected DLBCL morphology but retained the grade 1 (centrocytic or small cleaved cell) morphology [7], while clearly progressing from indolent to aggressive phenotype both in vitro and in vivo (SCID XT). CJ cells are not only DHL cells but also currently the only known centrocytic cell line, with a unique pathophysiology, suggesting that *MYC/BCL2* DHL is heterogeneous and may provide insights into pathophysiologic mechanisms such as large cell transformation of follicular lymphoma. Although we believe the RC cell line will be an excellent experimental tool to study *MYC/BCL2* double-hit lymphoma, other additional cell lines, for example to study *MYC/BCL6* double-hit lymphomas, will also be needed to better understand these less common DHL tumors.

In summary, in this study, we report the establishment and characterization of a novel *MYC/BCL2* DHL cell line with morphologic features of DLBCL, RC, that immunophenotypically and cytogenetically closely resembles the primary B cell tumor at diagnosis. We believe that the newly characterized DHL cell line will provide useful in vitro and in vivo models for translational and biological studies related to human DHL, which is refractory to current therapy and urgently needs novel therapeutic approaches.

## Materials and methods

### Cell culture

The University of Texas MD Anderson Cancer Center Satellite Tissue Bank provided the patient samples used for these studies. With informed consent from the patient, the collected primary cells were purified from ascites by Ficoll centrifugation (Ficoll-Paque Plus; GE Healthcare, Life Sciences, Piscataway, NJ), washed in phosphate-buffered saline twice, and resuspended in RPMI 1640 (Life Technologies, Grand Island, NY) containing 15 % heat-inactivated FBS, 2 mM glutamine, and 50 μg/mL gentamycin at a concentration of 5–10 × 10^6^ cells/mL (40 mL) in 75-cm^2^ flasks. Cultures were maintained at 37 °C in a humidified incubator with a 5 % CO_2_ atmosphere. The medium was exchanged every 3–5 days depending on the cell growth rate. The cells were examined daily using an inverted microscope and counted weekly with a standard hemocytometer using trypan blue dye exclusion. No external growth factors or stimulatory cytokines were added during the establishment of the RC cell line.

### Cell growth and viability assay

Cell viability was assessed using the CellTiter-Glo Luminescent Assay (Promega). Cells were plated in triplicates at 1–2 × 10^4^ cells/well in 96-well plate with increase concentrations of AZD8055 (Selleckchem) in 100 μl total volume. Cell viability was assessed at 72 h after treatment.

### Western blot analysis

Cell lysates were prepared and immunoblotted as previously described [29, 30].

### Flow cytometry

Eight-color flow cytometry analysis was performed with FACS Canto II instruments (BD Biosciences, San Jose, CA) using commercially available reagents on patient samples collected in ethylenediaminetetraacetic acid (EDTA) or cell line cells in culture medium. The cell population was gated using right-angle light scatter and CD45 expression. The panel of monoclonal antibodies used included those specific for CD3, CD4, CD5, CD8, CD10, CD11c, CD19, CD20, CD22, CD23, CD30, CD34 CD38, CD43, CD44, CD45, CD56, CD200, and surface kappa and lambda light chains. All antibodies were purchased from BD Biosciences. Data were analyzed using FCS Express software (De Novo Software, Los Angeles, CA). Antigen expression was scored as positive based on a significant shift in staining in comparison to a negative autofluorescence (empty channel) control.

### Conventional cytogenetic analysis

RC cells were stimulated with phytohemagglutinin for 72 h before conventional G-banded karyotyping was performed with metaphase cells derived from tumor cell cultures. Briefly, metaphase cells were obtained after hypotonic treatment and fixation with 3:1 methanol-acetic acid solution using automatic harvesting system. Cell suspensions derived from the automatic harvesting system were dropped onto cleaned slides. G-banding was performed after the slides were dried at 60 °C overnight. Chromosome analysis and karyotyping, after the use of Genetix metaphase automatic scanning system, were performed with the CytoVision system. Twenty metaphases were fully analyzed as per standard protocols.

Fluorescence in situ hybridization (FISH) was performed on interphase nuclei from the cell culture using a dual-color, break apart *MYC* probe and *IGH/BCL2* dual-color, dual-fusion translocation probes (Abbott Molecular, Des Plaines, IL), as described previously. The cutoff to define a positive result for rearrangement of *MYC*, and *IGH/BCL2* probe is 3.8 and 0.1 %, respectively. A total of 200 interphase cells were analyzed.

### Short tandem repeat DNA fingerprinting

Genomic DNA was isolated from the original tumor and the RC cell line using a Qiagen DNA purification kit (Valencia, CA). DNA fingerprinting of lymphoma cells was performed by the Institutional Characterized Cell Line core facility at MD Anderson using the STR method. Short tandem repeats are regions of microsatellite instability with defined tri- or tetra-nucleotide repeats that are located throughout the chromosomes. A PCR-based method using primers on non-repetitive flanking regions to generate PCR products of different sizes based on the number of repeats in the region was performed; the size of the products was determined by capillary electrophoresis. Extracted DNA was analyzed using the Power Plex 16HS System from Promega (Madison, WI). The relatedness of the original tumor and the RC cell line was determined by comparing the STR loci profiles of the respective samples.

### Epstein-Barr virus PCR amplification

EBV genotyping was performed by PCR using genomic DNA to amplify a common region of the *EBNA1* and *EBNA2* gene using a PCR kit from Promega with the following set of primers: EBNA1-F: GGT AGA AGG CCA TTT TTC CAC; EBNA1-R: CTC CAT CGT CAA AGC TGC AC; EBNA2-F: CAG GTA CAT GCC AAC AAC CTT; EBNA2-R: CCA ACA AAG ATT GTT AGT GGA AT. The PCR cycling conditions were as follows: 95 °C 2 min, 40 cycles of 94 °C 1 min, 60 °C 90 s, 72 °C 4 min, followed by 72 °C for 10 min [31]. The EBV-negative Mino and the EBV-positive Granta mantle cell lymphoma cell lines were used as negative and positive controls, respectively.

### Xeno-transplant of RC cells in severed combined immunodeficient (SCID) mice

All animal experiments were reviewed and approved by the MD Anderson Institutional Animal Care and Use Committee (IACUC). For in vivo studies, 6-week-old female immumodeficient NOD.Cg-Prkdc^scid^I12r_g_^tm1Wj1^/SzJ mice were purchased from Jackson Laboratories (Bar Harbor, ME) and housed under specific pathogen-free conditions at the SCID Mouse Barrier Facility at MD Anderson. RC cells (10 × 10^6^) were injected intraperitoneally into the mice using a 27-gauge needle.

### Reverse-phase protein array (RPPA)

The RPPA Core Facility at MD Anderson Cancer Center performed the RPPA analysis and antibody validation [32]. For total protein lysate preparation, media were removed, and cells were washed twice with ice-cold phosphate-buffered saline (PBS) containing complete protease and PhosSTOPphosphatase inhibitor cocktail tablets (Roche Applied Science, Mannheim, Germany) and 1 mM Na3VO4. Lysis buffer (1 % Triton X-100, 50 mMHEPES (pH 7.4), 150 mM NaCl, 1.5 mM MgCl2, 1 mM EGTA, 100 mM NaF, 10 mM NaPPi, 10 % glycerol, 1 mM PMSF, 1 mM Na3VO4, and 10 μg/mLaprotinin). Samples were vortexed frequently on ice and then centrifuged. Protein lysates were adjusted to a 1 μg/μL concentration, and a serial dilution of five concentrations was printed, with 10 % of the samples replicated for quality control (2470 Arrayer; Aushon Biosystems) on nitrocellulose-coated slides (Grace Bio-Labs). Immunostaining was performed using a DakoCytomation-catalyzed system and diaminobenzidine colorimetric reaction. Slides were scanned on a flatbed scanner to produce 16-bit tiff images. Spot intensities were analyzed and quantified using Array-Pro Analyzer to generate spot signal intensities. Relative protein levels for each sample were determined by interpolation of each dilution curve from the “standard curve” constructed by a script in R written Bioinformatics. All the data points were normalized for protein loading and transformed to linear values that can be used for bar graph. Normalized linear value was transformed to log2 value, and then median-centered for hierarchical cluster analysis and for heatmap generation. The heatmap was generated in Cluster 3.0 (http://cluster2.software.informer.com/3.0/) as a hierarchical cluster using Pearson correlation and a center metric. The resulting heatmap was visualized in Treeview (http://rana.lbl.gov/EisenSoftware.htm) and presented as a high resolution .bmp format. Two hundred eighty-five unique antibodies and four secondary antibody negative controls were analyzed.

## Abbreviations

DHL: double-hit lymphoma
DLBCL: diffuse large B cell lymphoma
EBV: Epstein-Barr virus
FBS: fetal bovine serum
FISH: fluorescence in situ hybridization
R-CHOP: rituximab immunotherapy and cyclophosphamide, doxorubicin, vincristine, and prednisone
RPPA: reverse-phase protein array
SCID: severe combined immunodeficiency
